# Population‐Level Burden of Dermatitis and the Effects of Moxibustion and Capsaicin on Pruritus and JAK/STAT Signaling in Atopic Dermatitis: A Multilevel Study

**DOI:** 10.1155/mi/4932652

**Published:** 2026-09-16

**Authors:** Yunhong Yang, Lihua Guo, Yunsong Yang, Han Tang, Hongjun Kuang, Lvping Lin, Yi Gou, Siyuan Li, Hong Zhao

**Affiliations:** ^1^ Shanghai University of Traditional Chinese Medicine, Shenzhen Hospital, Shenzhen, China, shutcm.edu.cn; ^2^ Shanghai University of Traditional Chinese Medicine, Shanghai, China, shutcm.edu.cn; ^3^ Shenzhen Luohu District Hospital of Traditional Chinese Medicine, Shenzhen, China; ^4^ Tianjin University of Science and Technology, Tianjin, China, tust.edu.cn

**Keywords:** atopic dermatitis, GBD, JAK/STAT signaling pathway, moxibustion, pruritus

## Abstract

**Purpose:**

This study comprised two complementary levels of investigation. First, we characterized the global burden, temporal trends, and socioeconomic inequalities of dermatitis as an aggregated disease category. Second, within this broader public‐health context, we investigated whether moxibustion and topical capsaicin alleviate pruritus and inflammatory skin damage in a rat model of atopic dermatitis (AD) and explored their potential convergent regulation of the Janus kinase (JAK)/STAT signaling pathway.

**Methods:**

The study comprised a population‐level contextual analysis and a disease‐specific experimental investigation. Age‐standardized incidence rates (ASIRs) and disability‐adjusted life years (DALYs) attributable to each risk factor for dermatitis were extracted and assessed from the Global Burden of Disease (GBD) database. The GBD component was used to characterize the broader public‐health burden of dermatitis and was not interpreted as an AD‐specific epidemiological analysis. An AD rat model was established using 2,4‐dinitrochlorobenzene (DNCB) in an acetone solution. Successfully modeled rats were randomly assigned to untreated model, mometasone furoate cream, moxibustion, or topical capsaicin groups. The standard pharmacological treatment group was included as a therapeutic efficacy benchmark, whereas capsaicin was used as a sensory‐neuron‐modulating active comparator for the exploratory analysis of downstream molecular convergence. Proteomic analysis was used to identify treatment‐associated pathways, and changes in IFN‐γ/JAK1/STAT1/STAT3 signaling components were subsequently assessed by western blotting, RT‐qPCR, and immunohistochemistry. In addition to skin‐lesion severity and scratching behavior, pruritus‐related neuroimmune mediators were evaluated. Serum IL‐31 concentrations were measured by ELISA. Cutaneous IL‐31 and TRPV1 expression was assessed by immunohistochemistry, and substance P immunoreactivity was evaluated by immunofluorescence. The relative mRNA expression of Trpv1, SP(Tac1), and CGRP in lesional skin was measured by RT‐qPCR.

**Results:**

The global incidence of dermatitis increased, with a higher burden in females. Significant inequalities in incidence and DALYs existed across countries of varying sociodemographic index (SDI) levels. At the experimental level, target intersection analysis identified 302 genes shared by capsaicin, AD, and pruritus, while lesional‐skin proteomics identified 257 common differentially expressed proteins. Exploratory proteomic enrichment identified JAK/STAT signaling as the most prominent candidate pathway in the enrichment bubble plot. AD model rats exhibited increased scratching, upregulated pruritus‐related mediators (IL‐31, TRPV1, substance P, and Trpv1/Tac1/CGRP transcripts), exacerbated skin lesions, histopathological damage, and a pro‐inflammatory shift (elevated IL‐1, IL‐4, IL‐6, TNF‐α, IFN‐γ; reduced IL‐10). Mometasone furoate predictably alleviated these AD‐like manifestations. Both moxibustion and capsaicin treatments reversed these trends, improving pathology, restoring spleen/thymus indices, and downregulating IFN‐γ expression and JAK1/STAT1/STAT3 phosphorylation.

**Conclusion:**

Aggregated dermatitis continues to impose a substantial and unevenly distributed global health burden. Within this broader public‐health context, the experimental findings indicate that moxibustion and topical capsaicin alleviate pruritus and inflammatory skin damage in rats with AD. These improvements were accompanied by attenuation of IFN‐γ/JAK1/STAT1/STAT3‐associated signaling, supporting the potential involvement of the JAK/STAT pathway in the observed treatment responses.

## 1. Introduction

Atopic dermatitis (AD) is a common itchy, inflammatory skin condition, with dry skin and itching being its classic symptoms [1]. Currently, AD has emerged as a significant health challenge that society must address, and it has placed a considerable economic burden on society [2]. AD demonstrates high clinical heterogeneity. Its etiology is not yet fully understood, and the pathophysiology is complex, involving interaction and crosstalk among epidermal barrier defects, skin microbiome dysbiosis, and immune dysregulation [3]. Clinical practice guidelines recognize corticosteroids as the primary and most effective treatment for AD, which can be employed in systemic therapy. However, large‐area or long‐term use increases the financial pressure on patients [4, 5]. From a clinical perspective, persistent pruritus is among the most difficult symptoms for patients to tolerate and markedly compromises their quality of life. Therefore, exploring effective, safe, and affordable therapeutic approaches to alleviate pruritus associated with AD is of significant clinical importance.

In the Global Burden of Disease (GBD) 2021 cause hierarchy, dermatitis is reported as an aggregated disease category encompassing AD, contact dermatitis, and seborrheic dermatitis [6]. Therefore, population‐level estimates for dermatitis provide a broad overview of the health burden associated with common inflammatory dermatoses but cannot be interpreted as epidemiological estimates specific to AD. In the present study, the GBD analysis was included to establish this broader public‐health context and to characterize temporal and socioeconomic disparities in dermatitis burden. AD was subsequently selected for disease‐specific experimental investigation because persistent pruritus, recurrent cutaneous inflammation, and immune dysregulation are central features of the disease and are directly relevant to the proposed therapeutic effects of moxibustion and capsaicin. Accordingly, the two components address complementary but distinct questions: the epidemiological analysis characterizes population‐level burden, whereas the animal study evaluates therapeutic efficacy and molecular mechanisms in AD.

Although not explicitly named in ancient medical texts, AD falls under the category of “Shi Chuang” (eczema) in Traditional Chinese Medicine (TCM). Historically, TCM physicians have consistently attributed the core pathogenesis of chronic eczema to a state of “root deficiency and superficial excess.” The condition manifests on the skin externally, yet its root cause lies internally. The convergence of internal and external pathogenic factors leads to its development, resulting in the characteristic clinical features of a “protracted course, frequent recurrence, and persistent, recalcitrant nature” [7]. Moxibustion is a convenient and economical traditional Chinese medical therapy. Research indicates that moxibustion can markedly alleviate pruritus associated with dermatological condition [8, 9]. According to the Expert Consensus on TCM Diagnosis and Treatment of Eczema (Shi Chuang), moxibustion has been recommended as a therapeutic option for chronic eczema. Its mechanism of action is attributed to its ability to dredge and regulate qi and blood as well as to unblock the meridian system. This facilitates the dispersal of internal damp‐heat and stagnant fire, thereby reinforcing healthy qi, eliminating pathogenic factors, and promoting recovery. Nevertheless, the precise mechanisms by which moxibustion alleviates pruritus in AD remain to be elucidated. Research indicates that topical capsaicin application can effectively alleviate acute pruritus [10, 11]. Capsaicin initially elicits a burning sensation upon application. However, with prolonged use, it can induce neuronal desensitization, which alleviates chronic pruritus. Topical capsaicin was selected as an active comparator because it represents a well‐characterized form of peripheral sensory modulation. Capsaicin chemically activates transient receptor potential vanilloid 1 (TRPV1), a polymodal ion channel responsive to capsaicin, noxious heat, acidic conditions, and several endogenous inflammatory mediators [12, 13]. Initial TRPV1 activation excites capsaicin‐sensitive sensory afferents and produces burning, pricking, and itching sensations. Repeated or sustained topical exposure may subsequently induce functional desensitization or inactivation of these sensory fibers, accompanied by reduced sensory responsiveness [14]. Moxibustion, in contrast, delivers controlled physical thermal stimulation to the skin and may additionally influence local blood perfusion, cellular stress responses, sensory afferent activity, and the cutaneous immune microenvironment [15]. Thus, the two interventions differ substantially in their proximal modes of action but may engage partially overlapping neurocutaneous processes. The comparison was therefore based on a neuroimmune convergence hypothesis. Both interventions are applied locally to lesional skin, influence peripheral sensory processing, and may alter the bidirectional communication among sensory nerve endings, keratinocytes, and immune cells that contributes to the itch–scratch–inflammation cycle in AD [16, 17]. Capsaicin was included as a mechanistically informative active comparator to determine whether moxibustion produced parallel improvements in pruritus and inflammation and whether the two interventions were associated with convergent downstream molecular changes. Therefore, this study performed an intersection analysis of genes related to capsaicin, AD, and pruritus. Subsequent bioinformatics analysis of the overlapping genes, combined with proteomic results from local lesional tissues in rats, revealed that the Janus kinase (JAK)/STAT signaling pathway may play a critical role.

JAKs are a family of intracellular nonreceptor tyrosine kinases that transduce cytokine‐mediated signals into nuclear responses via the JAK‐STAT pathway. The JAK/STAT signaling pathway mediates several critical biological processes, including cell proliferation, differentiation, apoptosis, and immunoregulation [4]. Upon ligand binding to transmembrane cytokine receptors (such as interferon receptors), JAKs are activated, leading to the phosphorylation and activation of STAT proteins. The activated STATs then translocate to the nucleus to regulate the transcription of target genes [5]. More than 50 cytokines signal through the JAK/STAT pathway to coordinate immune functions, mediate inflammatory responses, and facilitate tissue repair. The activity of this pathway is often upregulated in response to increased levels of pro‐inflammatory cytokines [18]. Immune dysregulation is a pivotal determinant of heightened environmental hypersensitivity and the development of inflammation in patients with AD [19].

Therefore, this study adopted a multilevel design. At the population level, we analyzed the temporal trends and socioeconomic inequalities of dermatitis using GBD 2021 data to define the broader public‐health context. At the disease‐specific level, we investigated and compared the antipruritic and anti‐inflammatory effects of moxibustion and topical capsaicin in dinitrochlorobenzene (DNCB)‐induced AD. The present study did not treat capsaicin as a direct surrogate for moxibustion. Instead, capsaicin was used as a topically administered, sensory‐neuron‐modulating active comparator. We first compared the effects of the two interventions on scratching behavior, skin lesions, histopathological injury, and inflammatory responses in DNCB‐induced AD. We then integrated candidate‐target screening with lesional‐skin proteomics to investigate whether these distinct interventions were associated with convergent downstream molecular pathways. Selected components of the IFN‐γ/JAK1/STAT1/STAT3 axis were subsequently assessed as pathway‐associated markers.

## 2. Materials and Methods

### 2.1. Population‐Level Contextual Analysis of the Global Burden of Dermatitis

#### 2.1.1. Data Sources and Case Definition

The present analysis used the GBD Level 3 aggregated category of dermatitis, which comprises AD, contact dermatitis, and seborrheic dermatitis. Population‐level data were obtained from the GBD Study 2021 [20, 21]. The epidemiological analysis was based on the Level 3 cause “Dermatitis,” which represents an aggregated category encompassing AD, contact dermatitis, and seborrheic dermatitis. No customized reclassification or aggregation of disease subtypes was performed. Because subtype‐specific estimates were not available in the dataset extracted for the present analysis, all incidence and disability‐adjusted life‐year estimates reported in this section refer to overall dermatitis.

Age‐standardized incidence rates (ASIRs) and disability‐adjusted life years (DALYs) were extracted at the global, regional, national, sex‐specific, and sociodemographic index (SDI) levels for the period 1990–2021. Estimates are reported per 100,000 population with their corresponding 95% uncertainty intervals. In the GBD, incidence represents the occurrence of new cases within the population at risk during a specified period and was used to characterize temporal changes in the population‐level occurrence of dermatitis.

#### 2.1.2. The SDI

The SDI is a composite measure of development based on income per capita, average educational attainment, and fertility among individuals younger than 25 years. Locations were classified into five SDI categories according to the GBD 2021 framework: high SDI, medium–high SDI, medium SDI, medium–low SDI, and low SDI.

#### 2.1.3. Temporal‐Trend Analysis

Temporal trends in ASIRs were evaluated using joinpoint regression. Annual percentage changes and average annual percentage changes were calculated with 95% confidence intervals. The Monte Carlo permutation method [22] was employed using randomly permuted datasets, with Bonferroni correction applied to maintain the overall asymptotic significance level. An increasing trend over a given period was identified if both the APC/average annual percent change (AAPC) estimate and the lower bound of its 95% CI were greater than 0. Conversely, a decreasing trend was identified if both the APC/AAPC estimate and the upper bound of its 95% CI were less than 0. Otherwise, the trend was considered stable.

### 2.2. Screening Disease Targets and Key Signaling Pathways Using Public Databases

This study systematically retrieved gene sets related to “atopic dermatitis,” “capsaicin,” and “pruritus” as core keywords from the GeneCards database. An intersection analysis was performed on these datasets to screen out overlapping candidate target genes. Subsequently, the obtained gene list was submitted to the DAVID database (https://davidbioinformatics.nih.gov) for Gene Ontology (GO) functional annotation and Kyoto Encyclopedia of Genes and Genomes (KEGG) pathway enrichment analysis, and the corresponding results were downloaded. Finally, the enrichment results were visualized using the online bioinformatics platform (https://www.bioinformatics.com.cn), which supports enrichment analysis and the generation of various scientific charts, to reveal the potential functions and associations of these common genes in biological processes, molecular functions, cellular components, and key signaling pathways.

### 2.3. Animal Study

#### 2.3.1. Experimental Animals and Grouping

Fifty‐six SPF‐grade healthy female SD rats weighing 180–220 g were supplied by Zhuhai Bestone Biotechnology Co., Ltd. [License Number: SCXK(Yue)2020‐0051]. All animals in this study were housed in the laboratory of Guangzhou Huateng Biomedicine Co., Ltd. The housing conditions were strictly controlled as follows: temperature at 20–26°C, relative humidity at 40%–70%, a pressure differential maintained above 10 Pa, a 12‐h light/dark cycle, an air exchange rate of no less than 15 times per hour, with free access to food and water. This experiment has been approved by the Laboratory Animal Use and Management Committee of Guangzhou Huaten Bio‐Pharmaceutical Co., Ltd., IACUC Number: C202403‐6.

#### 2.3.2. Main Reagents and Instruments

The main reagents and consumables used in the experiment were as follows: pure moxibustion sticks (Nanyang Wanbei Moxa Wool Factory), capsaicin cream (Fujian Lifike Pharmaceutical Co., Ltd., Fujian, China; National Medicine Permit No. H20080089), mometasone furoate cream (Shanghai Xinya Pharmaceutical Minhang Co., Ltd., China; National Medicine Permit No. H19991418), antibodies against interferon gamma (Affinity Biosciences, Cat# DF6045, RRID:AB_2838015), JAK1 (Affinity Biosciences, Cat# AF5012, RRID:AB_2834933), and STAT1 (Affinity Biosciences, Cat# AF6300, RRID:AB_2835149); substance P polyclonal antibody (Proteintech Cat#: Ag4790), TRPV1 polyclonal antibody (Proteintech Cat#: Ag185830; IL‐31 antibody (Boster Biological Technology, Wuhan, China. Cat#A08339‐1), goat anti‐rabbit IgG (Proteintech, Cat# SA00001‐2) and goat anti‐mouse IgG (Proteintech, Cat# SA00001‐1), β‐actin antibody (Beijing Zhuangmeng International Bio‐gene Technology Co., Ltd., Cat# A0101), 96T ELISA kits for rat IFN‐γ, TNF‐α, IL‐1, and IL‐4 (Dousail Biotechnology Co., Ltd., Cat# DRE20041, DRE20040, DRE20113, DRE20093, respectively), Rat IL‐31 (Interleukin‐31) ELISA Kit (FineTest, Cat#ER1716), Mayer’s hematoxylin staining solution (Solarbio, Cat# G1080), and eosin staining solution (Solarbio, Cat# G1100); and 2,4‐dinitrochlorobenzene (Sigma, USA).

The primary instruments included a Spectra Max Plus 384 microplate reader (Molecular Devices, USA), a qTOWER high‐speed real‐time quantitative PCR system (Analytik Jena AG, Germany), a Centrifuge 5424 R high‐speed refrigerated centrifuge (Eppendorf, Germany), an ABS‐MS‐078 constant temperature mixer (Hefei Aibensen Scientific Instrument Co., Ltd., China), and the ImageJ image analysis system (Media Cybernetics, USA).

#### 2.3.3. DNCB‐Induced AD Rat Model

According to relevant literature [8] and the “Specification for Preparation of Eczema Animal Models” [23], an AD rat model was established by induction with 2,4‐DNCB [24, 25]. Two areas (A and B), each ~3.0 cm × 3.0 cm, were delineated on the shaved backs of rats in the moxibustion and model groups. Sensitization phase (Day 1): 200 μL of a freshly prepared 7% DNCB solution in acetone was evenly applied topically to area A using a pipette. Challenge phase: 7 days later, an equal volume of 0.1% DNCB in acetone was applied to area B for the first challenge. This challenge was repeated every 3 days for a total of six times. Rats in the blank control group were not modeled; instead, an equivalent volume of normal saline was applied to the corresponding dorsal area at the same time points. During the modeling period, the skin lesion status was assessed daily with reference to the literature [26] and the Eczema Area and Severity Index (EASI) scoring criteria [27, 28]. Visually observe whether rats exhibit varying degrees of itching, skin lesions, erythema, reduced coat luster, and decreased activity.

#### 2.3.4. Intervention Methods and Grouping

Using a random number table, the rats were randomly divided into the following five groups:

Control group (blank group, *n* = 10): no intervention was applied. Animals were maintained under standard conditions until the end of the experiment. AD group (*n* = 12): following successful model establishment, animals were subjected to grasping and restraint daily, matching the duration and intensity applied to the moxibustion group but without receiving treatment.

Mometasone furoate group (MF group, *n* = 10): following successful model establishment, mometasone furoate cream (0.1%; 5 g/tube; Shanghai Xinya Pharmaceutical Minhang Co., Ltd., China) was evenly applied to the lesional dorsal skin at a dose of 1 g/kg every other day for two consecutive weeks. This group was included as a standard topical corticosteroid reference to provide a benchmark for evaluating the therapeutic efficacy of moxibustion and capsaicin.

Moxibustion group (Mox group, *n* = 12): after successful model establishment, rats were placed in a prone position and secured to a board with adhesive tape, including head fixation. According to Experimental Acupuncture Science [29], the local skin lesion area was selected as the “Ashi point” for intervention. A moxa stick was ignited and placed 1.5 cm above the target skin lesion area. The distance was maintained using manual positioning by the same trained operator. As the moxa stick burned and shortened, its position was adjusted to maintain the specified distance and target skin‐surface temperature. Ash was removed when necessary to maintain stable combustion. Each moxibustion session lasted 30 min, during which the local skin temperature was monitored and maintained at 43 ± 1°C using a thermometer. The operator also assessed the temperature with the fingertip and observed the dorsal skin for blister formation or erythema. The thermometer reading, rather than subjective heat perception, was used as the primary criterion for thermal standardization. Fingertip assessment by the operator was used only as an auxiliary safety check. All moxibustion procedures were performed by the same trained operators who received standardized training using moxa sticks of the same specification and manufacturing batch. The rats were treated in the same experimental room without direct airflow over the treatment area. The lesion area, animal position, moxa‐to‐skin orientation, treatment duration, target temperature, and treatment frequency were kept consistent across animals. The dorsal skin was continuously observed for excessive erythema, blistering, or other evidence of thermal injury. Mild transient erythema without blistering was considered an acceptable local response. Treatment was interrupted if marked erythema or blistering occurred. No thermal burns or blistering were observed during the study. Moxibustion was administered every other day for two consecutive weeks.

Capsaicin group (Cap, *n* = 12): following successful model establishment, a commercially available capsaicin cream was applied evenly to the lesional dorsal skin. The cream contained 22.5 mg of total capsaicinoids per 30 g of the formulation, corresponding to a concentration of 0.075% (w/w). The dosage of the ointment was 1 g/kg (based on the ointment weight). The intervention was administered every other day for two consecutive weeks. During each treatment session, the rats underwent grasping and restraint procedures identical to those applied to the moxibustion group in terms of duration and intensity.

A total of 56 rats were included in the experiment. The mometasone furoate and capsaicin groups served different purposes in the experimental design. Mometasone furoate, a topical corticosteroid used for the treatment of inflammatory and pruritic dermatoses, was included as a standard pharmacological reference for therapeutic efficacy. Capsaicin was included as a sensory‐neuron‐modulating active comparator to explore potential downstream molecular convergence with moxibustion.

#### 2.3.5. Randomization and Blinding

Animals were allocated to the experimental groups using a random‐number table. Because the interventions were visibly different and required distinct administration procedures, the investigators responsible for delivering moxibustion, mometasone furoate cream, and capsaicin cream could not be blinded to treatment allocation.

The investigators responsible for skin lesion scoring, histopathological evaluation, and ELISA measurements were blinded to the treatment allocation during the outcome assessment. Group information was masked during these evaluations and was not used in assigning scores or recording the assay results.

#### 2.3.6. Sample Collection

At the predetermined terminal time point, rats were deeply anesthetized with 2% pentobarbital sodium (45 mg/kg, intraperitoneally). The absence of response to a painful stimulus (e.g., a toe pinch) was confirmed to ensure an adequate depth of anesthesia. Following this, death was confirmed by the cessation of spontaneous breathing and heartbeat, and fixed, dilated pupils. The entire euthanasia procedure was conducted in strict accordance with the American Veterinary Medical Association (AVMA) Guidelines for the Euthanasia of Animals. Blood was collected via the abdominal aorta, and the animals were euthanized. The blood samples were centrifuged to separate the serum, and the supernatant was stored at −20°C for subsequent analysis. Subsequently, an ~3 cm × 3 cm area of shaved skin was excised and divided into four pieces along perpendicular vertical and horizontal lines. The two diagonal pieces were combined into one sample. One portion of the skin sample was placed in a cryotube, rapidly frozen in liquid nitrogen, and stored at −80°C for further use; the other portion was immersed in 4% paraformaldehyde for fixation. Meanwhile, the thymus and spleen were dissected, and the surrounding fascia and adipose tissue were carefully removed. After blotting residual blood with filter paper, the thymus and spleen were weighed (mg), and the thymus index (TI) and spleen index (SI) were calculated.

### 2.4. Proteomics Analysis

#### 2.4.1. Study Design and Sample Preparation

Proteomic profiling was conducted as an exploratory, hypothesis‐generating analysis. Three animals were randomly selected from each of the control, AD, Mox, and Cap groups. Each animal provided one independent biological replicate, and samples from different animals were not pooled. The MF group was not included in the proteomic profiling because the analysis was prespecified to investigate potential downstream molecular convergence between moxibustion and capsaicin.

Approximately 20 mg of frozen lesional skin was lysed in 200 μL of buffer containing 8 M urea, 1% SDS, and protease inhibitors. After centrifugation, the protein concentration was determined using the BCA assay. A 100‐μg protein aliquot from each sample was reduced with 10 mM TCEP, alkylated with 40 mM iodoacetamide, precipitated with prechilled acetone, and digested overnight with trypsin at an enzyme‐to‐protein ratio of 1:50.

Digested peptides were desalted using HLB solid‐phase extraction cartridges, dried by vacuum centrifugation, and quantified using the Thermo Fisher peptide quantification kit (Catalog Number: 23275).

#### 2.4.2. Liquid Chromatography–Mass Spectrometry

Equal amounts of peptides from each biological sample were separated using a Vanquish Neo UHPLC system equipped with an ES906 analytical column (150 μm × 15 cm; Thermo Fisher Scientific). Mobile phase A consisted of 0.1% formic acid and 2% acetonitrile in water, whereas mobile phase B consisted of 0.1% formic acid and 80% acetonitrile in water. Peptides were separated using a 60‐min gradient at a flow rate of 500 nL/min.

Mass‐spectrometric analysis was performed using an Orbitrap Astral instrument operated in the data‐independent acquisition mode with positive‐ion detection. The ion‐source voltage was 1.5 kV, and the full‐scan mass range was 100–1700 m/z.

### 2.5. Outcome Assessments

#### 2.5.1. Skin‐Lesion Severity and Scratching Behavior

Based on the EASI score, the local edema, scratches, erythema, crusting, and lichenification of the rat skin lesions were observed. The detailed criteria for skin lesion scoring were as follows: ① edema: absent, mild, moderate, and severe edema were scored as 0, 1, 2, and 3 points, respectively. ② Excoriation: absence of excoriation was scored as 0, and presence was scored as 1 point. ③ Erythema and crusting: no erythema was scored as 0, mild erythema as 1, marked erythema without crusting as 2, severe erythema with mild crusting as 3, and severe erythema with severe crusting as 4 points. ④ Lichenification: Absence of lichenification was scored as 0, while mild, moderate, and severe lichenification were scored as 1, 2, and 3 points, respectively.

#### 2.5.2. Itching in Rats

Following each intervention, itching was assessed by observing the number of times rats licked or scratched the itchy area within 10 min.

#### 2.5.3. Body Weight and Thymus and Spleen Indices

The SI and TI were calculated. The formulas for the SI and TI are as follows: SI = spleen weight (mg)/body weight (g) and TI = thymus weight (mg)/body weight (g).

#### 2.5.4. ELISA Detection of Serum Levels of Inflammatory Cytokines and Chemokines

Serum cytokine concentrations were measured using commercial rat ELISA kits according to the manufacturers’ instructions. Samples were analyzed in duplicate, and concentrations were calculated from standard curves generated on the same plate. Concentrations were calculated from plate‐specific standard curves using a four‐parameter logistic regression. Technical replicates were averaged to obtain one value per animal. Statistical analysis was performed to determine the concentrations of IFN‐γ, TNF‐α, IL‐1, IL‐4, IL‐6, IL‐10, and IL‐31 in rat serum.

#### 2.5.5. Histopathological Evaluation

Skin tissue was placed in 4% paraformaldehyde solution for intermediate fixation. After dehydration and xylene clearing, the tissue was embedded in paraffin. Sections were stained with hematoxylin and eosin using standard procedures and examined under a light microscope. Histopathological evaluation focused on epidermal thickening, dermal inflammatory‐cell infiltration, edema, and other predefined AD‐like changes. Three sections per animal and three fields per section were evaluated. Multiple fields from the same animal were summarized to obtain one animal‐level result.

#### 2.5.6. RT‐qPCR

Total RNA was extracted from frozen lesional skin using the TRIzol reagent. RNA concentration and purity were assessed using an instrument, and samples with an A260/A280 ratio of 1.8–2.0 were used for reverse transcription. cDNA was synthesized using the Evo M‐MLV Reverse Transcription Kit according to the manufacturer’s instructions. The reaction mixture was dispensed into qPCR plate wells, sealed, and placed in the qPCR instrument for amplification. Finally, the data were collected and analyzed. The relative expression of IFN‐γ(Ifng), Trpv1, SP(Tac1), and CGRP was measured. Primer sequences are provided in Supporting Information 1: Table S2.

#### 2.5.7. Western Blotting

Western blot analysis was performed according to standard procedures. In brief, total protein was extracted from tissues, and equal amounts of protein samples were separated by 10% SDS‐polyacrylamide gel electrophoresis. The separated proteins were then electrophoretically transferred onto polyvinylidene fluoride membranes. After blocking with 5% skimmed milk at room temperature for 1 h, the membranes were incubated with appropriate primary antibodies at 4°C overnight. Following washing, the membranes were incubated with horseradish peroxidase‐conjugated secondary antibodies at room temperature for 1 h. Finally, the antigen‐antibody complexes were visualized using a chemiluminescence reagent (Thermo Scientific, Rockford, IL, USA) and quantified by densitometric analysis with ImageJ software.

#### 2.5.8. Immunohistochemistry

Paraffin‐embedded skin sections were cut at 5 μm, subjected to antigen retrieval and endogenous‐peroxidase blocking, and incubated with antibodies against IFN‐γ, IL‐31, and TRPV1. Immunoreactivity was visualized using DAB and counterstained with hematoxylin. Finally, images were observed and captured under an upright light microscope. Five random fields of view were selected per section, and positive expression was defined by the presence of brownish deposits in the nucleus and/or cytoplasm.

#### 2.5.9. Immunofluorescence

Paraffin‐embedded skin sections were deparaffinized, rehydrated, and subjected to antigen retrieval. After blocking, sections were incubated with anti‐substance P primary antibody overnight at 4°C, followed by a fluorophore‐conjugated secondary antibody and DAPI counterstaining. Fluorescence images were acquired under identical settings, and substance P immunoreactivity was quantified using ImageJ. Five fields per section were averaged per animal, with background fluorescence corrected accordingly.

### 2.6. Data Processing and Normalization

Technical replicates and multiple microscopic fields obtained from the same animal were averaged before inferential statistical analysis and were not treated as independent biological replicates.

ELISA concentrations were calculated using plate‐specific standard curves. RT‐qPCR data were normalized to β‐actin and analyzed using the 2^−ΔΔCt^ method. Western blot signals for total proteins were normalized to β‐actin, whereas phosphorylated proteins were normalized to their corresponding total proteins. Immunohistochemical and immunofluorescence measurements were quantified using the actual metric under constant image‐acquisition and ImageJ analysis settings. Spleen and thymus weights were normalized to terminal body weight and expressed as mg/g.

### 2.7. Bioinformatics Analysis

All data were analyzed using the *t*‐test function in *R* to calculate the significance (*p*‐value) and fold change (FC) of intergroup differences. For each quantified protein, differences in protein abundance among the experimental groups were initially assessed using one‐way analysis of variance (ANOVA). When the overall ANOVA indicated a significant group effect, Tukey’s multiple‐comparisons test was performed for post hoc pairwise comparisons.To account for the large number of proteins tested simultaneously, the resulting protein‐level *p*‐values were adjusted using the Benjamini–Hochberg false discovery rate procedure. Proteins with an adjusted *p*‐value < 0.05 and a FC > 1.2 or < 0.833 were considered significantly differentially abundant.

GO annotation and functional clustering analysis were performed on the differentially expressed proteins using the GO database (http://geneontology.org/) for biological processes, cellular components, and molecular functions. Concurrently, the KEGG database (http://www.genome.jp/kegg/) was utilized to analyze the signaling pathways involved in the differentially expressed proteins.

### 2.8. Statistical Analysis

The ASIR is expressed as the number of cases per 100,000 population, along with its 95% UI. The APC and AAPC are presented as estimates with their 95% CIs. All analyses and visualizations were conducted using R software (Version 4.4.1). Additionally, SPSS software (Version 25.0) was used for data management and statistical analysis, GraphPad Prism (Version 9.5) was employed for graph plotting, and ImageJ software was utilized for experimental image analysis. For comparisons of protein expression levels between groups, a two‐tailed Student’s *t*‐test was applied. Measurement data conforming to a normal distribution are expressed as the mean ± standard deviation (*x* ± *s*). Differences among three or more groups were compared using one‐way ANOVA. If the assumptions of homogeneity of variances and normal distribution were met, Tukey’s post hoc test was used for pairwise comparisons. If these assumptions were violated, the nonparametric Kruskal–Wallis test was applied, followed by Dunn’s test for multiple comparisons correction. The statistical significance threshold was set at *α* = 0.05. *p*‐values adjusted for multiple comparisons are denoted with asterisks:  ^∗^ *p*  < 0.05,  ^∗∗^ *p*  < 0.01, ^∗∗∗^ *p*  < 0.001, and  ^∗∗∗∗^ *p*  < 0.0001.

## 3. Results

### 3.1. Population‐Level Burden and Socioeconomic Inequalities of Aggregated Dermatitis

**Figure 1:**
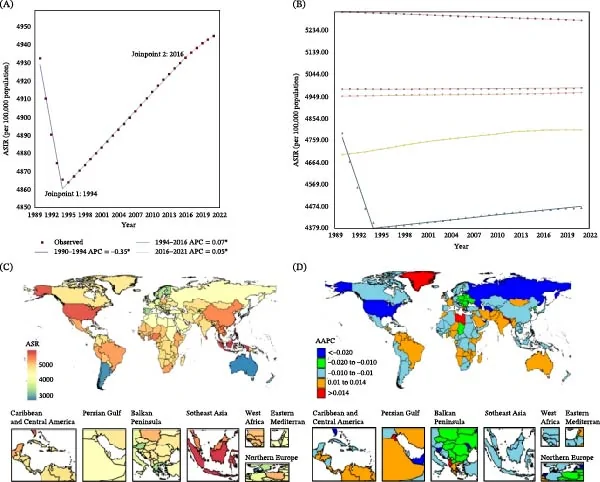
Global trends and burden of dermatitis incidence from 1990 to 2021: an analysis based on socioeconomic development. (A) Temporal trends in the global ASIR of dermatitis from 1990 to 2021. (B) Analysis of dermatitis ASIR across five regions with different socioeconomic status, as defined by the SDI. (C) World map of ASIR of dermatitis in 2021. (D) World map of AAPCs in ASIR of dermatitis in 2021. AAPC, average annual percent change; APC, annual percentage changes; ASIRs, age‐standardized incidence rates; SDI, sociodemographic index.  ^∗^Indicates that the annual percent change (APC) is significantly different from zero at the alpha = 0.05 level. Final selected model: 2 joinpoints.

Globally, the total number of individuals with dermatitis increased by ~60%, from about 250 million in 1990 to around 400 million in 2021. The ASIR rose slightly from 4932 per 100,000 population in 1990–4945 per 100,000 in 2021. Between 1990 and 2021, the global ASIR for dermatitis showed a downward trend before 1994, followed by a significant upward trend thereafter (AAPC = 0.011%, 95% CI: 0.008–0.013, *p*  < 0.001) (Table 1 and Supporting Information 2: Table S1). The most notable changes occurred during the periods 1990–1994 (APC = −0.35%), 1994–2016 (APC = 0.07%), and 2016–2021 (APC = 0.05%) (Figure 1A).

**Table 1:** Age‐standardized incidence and AAPC of dermatitis at global and regional level, 1990–2021.

Category	Number of people with dermatitis in 1990 (000s)	Age‐standardized rate in 1990 (per 100,000)	Number of people with dermatitis in 2021 (000s)	Age‐standardized rate in 2021 (per 100,000)	AAPC (95% CI)
Global	248056 (218594–284716)	4932 (4335–5692)	405022 (355573–467256)	4945 (4355–5690)	0.011 (0.008–0.013)
Sex					
Female	132692 (116347–153291)	5273 (4612–6119)	216357 (188914–250560	5252 (4609–6071)	−0.01 (−0.013 to −0.007)
Male	115364 (102146–131643)	4594 (4067–5256)	188665 (166213–216697	4636 (4106–5301)	0.033 (0.031–0.036)
SDI level					
High SDI	44654 (39453–51058)	4789 (4258–5432)	53919 (47791–61546)	4467 (3991–5028)	−0.207 (−0.218 to −0.197)
High‐middle SDI	50759 (44157–59212)	4697 (4108–5457)	72225 (62468–84938)	4804 (4200–5582)	0.072 (0.071–0.074)
Middle SDI	82700 (71891–95849)	5313 (4624–6203)	138606 (119988–162433)	5277 (4601–6141)	−0.022 (−0.023 to −0.021)
Low‐middle SDI	49222 (43740–55845)	4950 (4353–5694)	92053 (81345–105186)	4966 (4368–5708)	0.01 (0.01–0.01)
Low SDI	20487 (18338–22957)	4980 (4448–5648)	47896 (42787–53747)	4986 (4455–5654)	0.003 (0.002–0.005)

Abbreviation: AAPC, average annual percent change.

The burden of dermatitis was observed to be highest in the medium SDI category, followed by low SDI, middle–low SDI, middle–high SDI, and finally high SDI (Figure 1B).The ASR of dermatitis in the population declined in countries with high and medium SDI in the period 1990–2021, with an AAPC of −0.207 % and −0.022 %, respectively. The ASR of dermatitis increased in the other SDI subgroups, especially in countries with high and medium SDI (AAPC 0.072 %). The ASIR of dermatitis was highest in countries with a high SDI, with an AAPC of 0.078%, which is 26 times the growth rate observed in countries with a low SDI, where the AAPC was 0.003% (Supporting Information 2: Table S1).

The global ASIR increased for both males and females, rising from 12,000 per million to 19,000 per million in males, and from 13,000 per million to 22,000 per million in females. The incidence of dermatitis showed an increasing trend in males, while females exhibited a decreasing trend (AAPC of 0.033% vs. −0.01%) (Table 1 and Supporting Information 3: Figure S1). The reduction in DALYs attributable to dermatitis was less pronounced in global males compared to females, with an annual average trend of −0.234%. Notably, in high SDI countries, the decrease in DALYs for males was more significant than for females, showing annual average trends of −0.182% for males and −0.161% for females. In medium–high and medium‐SDI countries, DALYs are decreasing for women, while the opposite is true for men. For the rest of the SDI countries, gender differences were consistent with global trends (Supporting Information 1: Table S2 and Supporting Information 3: Figure S1). However, for every 100,000 people between 1990 and 2021, the DALYs for dermatitis were significantly higher in women than in men. This gender difference was consistent across all SDI, with women having a significantly higher burden due to developing dermatitis than men (Supporting Information 4: Figure S2).

The highest incidence of dermatitis was concentrated in Southeast Asia, while the lowest incidence was observed in Australia, Argentina, Chile, and Uruguay (Figure 1C). Notably, the incidence of dermatitis in Southeast Asia has shown a declining trend. Conversely, the countries with the fastest‐growing incidence rates are located near Greenland, Libya, and Nepal (Figure 1D).

These population‐level findings demonstrate that dermatitis, considered as an aggregated disease category, remains a substantial and unequally distributed global health concern. However, the GBD data do not distinguish the therapeutic mechanisms of individual dermatitis subtypes and cannot establish an AD‐specific mechanistic hypothesis. Therefore, the following experimental component addresses a separate but complementary question: whether moxibustion and capsaicin can alleviate pruritus and inflammatory damage in AD and whether their effects converge on common immune‐inflammatory signaling pathways.

### 3.2. Key Targets of Capsaicin in AD Pruritus

**Figure 2:**
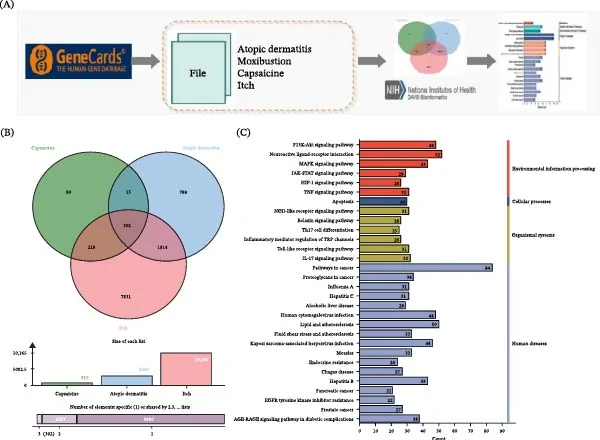
Identification of key targets for capsaicin in alleviating atopic dermatitis‐associated pruritus. (A) Flowchart of the screening process for key target genes. (B) Venn diagram illustrating the overlap among genes related to capsaicin, atopic dermatitis, and pruritus. Green circle: genes related to capsaicin; blue circle: genes related to atopic dermatitis; red circle: genes related to pruritus; overlapping area: number of genes common to all three sets. (C) Enriched molecular function analysis of the overlapping target genes. The top 30 most significant functions are displayed and presented by category.

Intersection analysis revealed 302 genes common to capsaicin, AD, and pruritus (Figure 2A). KEGG pathway enrichment analysis performed using the DAVID database identified the top 30 most significant pathways (Figure 2B). These included the PI3K‐Akt signaling pathway, MAPK signaling pathway, JAK‐STAT signaling pathway, and NOD‐like receptor signaling pathway, among others. These findings suggest that the antipruritic effect of capsaicin on AD may be mediated through the modulation of these signaling pathways.

### 3.3. Animal Experiments

#### 3.3.1. Mometasone Furoate Cream, Moxibustion, and Capsaicin Effectively Improve the Pathological State of 2,4‐DNCB‐Induced AD and Alleviate Itching Symptoms

All 56 animals completed the entire experimental protocol. No animals died during modeling or intervention, no animals were excluded because of modeling failure, adverse events, protocol deviation, or sample‐quality problems, and no animals were lost to follow‐up. Therefore, all 56 animals were included in the final analysis of the prespecified experimental outcomes.

**Figure 3:**
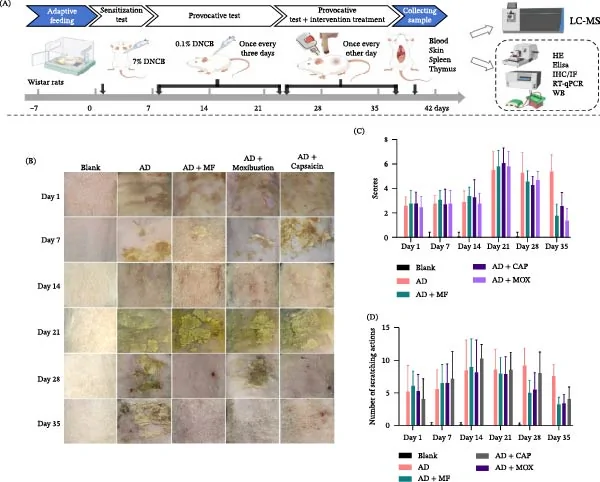
The mometasone furoate cream, moxibustion, and capsaicin ameliorated DNCB‐induced AD‐like skin lesions and scratching behavior. (A) Flowchart of the in vivo experimental procedures, including model establishment, intervention, and tissue collection. (B) Macroscopic images of local skin lesions recorded weekly, showing pathological features such as erythema, edema, crusting, and lichenification throughout the modeling period. (C) EASI scores. Data are presented as the mean ± standard deviation (SD), showing the lesion scores at weekly intervals. (D) Scratching frequency in rats. Observations were conducted after each intervention to assess pruritus. Data are presented as the mean ± standard deviation (SD), showing changes at weekly intervals. AD, atopic dermatitis; CAP, capsaicin; EASI, eczema area and severity index; MF, mometasone furoate cream; MOX, moxibustion.

Animals assigned to the AD and intervention groups underwent DNCB modeling, whereas animals in the blank control group did not receive DNCB. Following successful modeling, rats exhibited poor mental states, becoming easily startled and restless. Compared to the blank control group, distinct scratch marks or bite marks appeared on and near the skin lesions. The 2,4‐DNCB‐induced AD in rats exhibited sensitive dorsal skin, successfully mimicking the clinical features of AD. Visible pathological changes in the lesions included erythema, crusting, and lichenification. After modeling, rat skin lesion scores significantly increased and maintained the pathological conditions of disease lesions under provocation tests. However, mometasone furoate cream, moxibustion, and capsaicin improved pathological conditions and alleviated itching. Visible macroscopic examination revealed that after 2 weeks of treatment, skin in moxibustion‐treated and mometasone furoate cream‐treated rats returned to normal, while capsaicin‐treated rats still exhibited mild symptoms (Figure 3B). During the modeling period, both skin lesion scores and itching frequency were significantly higher in AD rats than in the blank control group. Following treatment, a decreasing trend in skin lesion severity was observed, accompanied by a reduced itching frequency (Figures 3C,D).

#### 3.3.2. Mometasone Furoate Cream, Moxibustion, and Capsaicin Improve Inflammatory Damage in AD Rats

**Figure 4:**
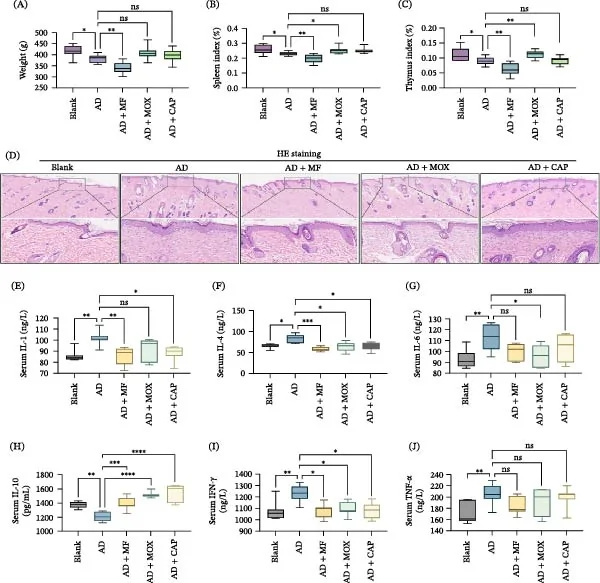
Comparison of the mometasone furoate cream, moxibustion, and capsaicin on histopathological and inflammatory outcomes. (A) Body weight (g) of rats in each group after the intervention. (B) Spleen index (mg/g) of rats. (C) Thymus index (mg/g) of rats. (D) Morphological changes and inflammatory damage in local lesional tissue assessed by H&E staining (4X, 20X). (E–J) Expression levels of IL‐1, IL‐4, IL‐6, IL‐10, IFN‐γ, and TNF‐α in serum from each group of rats, as determined by enzyme‐linked immunosorbent assay (ELISA). Data are presented as the mean ± standard deviation (SD) from biological replicates (*n* = 6), with each sample measured in triplicate (technical replicates).  ^∗^ *p*  < 0.05,  ^∗∗^ *p*  < 0.01,  ^∗∗∗^ *p*  < 0.001,  ^∗∗∗∗^ *p*  < 0.0001. AD, atopic dermatitis; CAP, capsaicin; IFN‐γ, interferon‐gamma; IL‐1, interleukin 1; IL‐4, interleukin 4; IL‐6, interleukin 6; IL‐10, interleukin 10; MOX, moxibustion; TNF‐α, tumor necrosis factor‐alpha.

Compared to the blank group, rats successfully modeled exhibited a significant decrease in body weight. However, rats treated with moxibustion showed a trend toward weight gain (Figure 4A). Spleen and thymus indices serve as indirect indicators for assessing immune system health and function. The SI is typically calculated as the ratio of the spleen weight to the mouse body weight. The TI is the ratio of thymus weight to the mouse body weight. Results showed that compared to the blank group, both the spleen and thymus indices in the model group exhibited a downward trend, while they increased significantly after moxibustion treatment. Mice in the capsaicin intervention group exhibited modest increases in body weight, SI, and TI, though these changes were not statistically significant. In contrast, mice in the mometasone furoate cream intervention group demonstrated a significant decline in all three parameters (Figure 4B,C).

Histological examination by H&E staining revealed a normal skin structure without significant inflammatory cell infiltration or hyperkeratosis in the control group. In contrast, the AD group exhibited marked epidermal and dermal thickening, acanthosis, and substantial inflammatory cell infiltration in the lesion areas. Compared with the AD group, the moxibustion group showed significant amelioration of these pathological features, with markedly reduced inflammatory cell infiltration and a histological appearance similar to that of the blank group (Figure 4D).

Analysis of serum inflammatory cytokines demonstrated that the levels of pro‐inflammatory cytokines (IL‐1, IL‐4, TNF‐α, and IFN‐γ) were significantly elevated, while the anti‐inflammatory cytokine IL‐10 was slightly decreased in the AD group compared with the control group. After moxibustion treatment, the levels of these pro‐inflammatory cytokines were markedly reduced, accompanied by an increase in IL‐10 (Figure 4E–J). Of note, the capsaicin and mometasone furoate cream groups showed comparable trends to the moxibustion group across all measured parameters. These findings suggest that the mechanism by which moxibustion alleviates AD may involve anti‐inflammatory effects, suppression of excessive immune responses, and modulation of the Th1/Th2 balance.

#### 3.3.3. Proteomic and Expression Analyses Identify JAK/STAT Signaling as a Treatment‐Associated Pathway

To elucidate the mechanism of action of moxibustion in treating AD, we conducted a proteomic analysis of local lesional skin tissues from rats in the blank, AD, moxibustion, and capsaicin groups. By comparing the AD group with the blank, moxibustion, and capsaicin groups, respectively, differentially expressed proteins were screened and intersected, resulting in 257 common differentially expressed proteins. KEGG analysis identified multiple candidate pathways associated with the treatment‐responsive protein set. Among the pathways shown in the enrichment bubble plot, JAK/STAT signaling exhibited the most prominent enrichment signal and was therefore ranked as the leading candidate pathway for targeted follow‐up. Other enriched pathways were retained as exploratory candidates; however, JAK/STAT signaling was prioritized because of its leading enrichment result, its additional support from the capsaicin‐AD‐pruritus target‐intersection analysis, and its biological relevance to cytokine‐mediated cutaneous inflammation.

**Figure 5:**
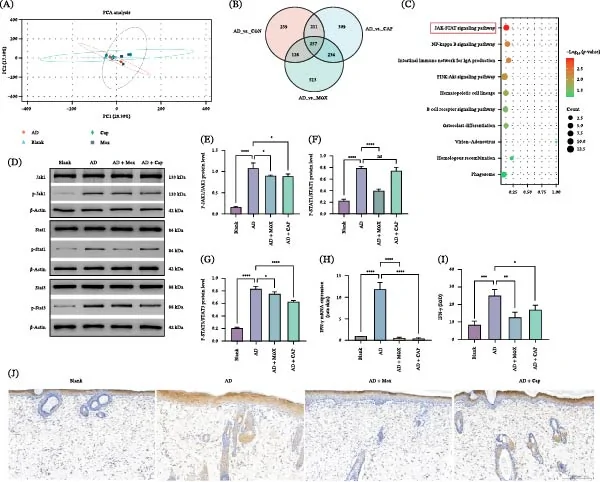
Effects of moxibustion and capsaicin on the JAK/STAT signaling pathway. (A) Principal component analysis (PCA) of sample expression profiles. PC1 and PC2 explain 28.30% and 17.30% of the expression variance, respectively. Points with different colors and shapes represent the four experimental groups: AD group (red dots), blank group (light blue triangles), capsaicin group (dark green diamonds), and moxibustion group (dark blue squares). The ellipses indicate the 95% confidence interval for each group. (B) Venn diagram showing the intersection of differentially expressed genes (DEGs). Red circle: DEGs from AD vs. blank comparison; blue circle: DEGs from AD vs. capsaicin comparison; green circle: DEGs from model vs. moxibustion comparison; overlapping area: number of genes common to all three comparisons. (C) Molecular function enrichment analysis of the overlapping target genes, displaying the top 10 most significant functions. (D–G) Western blot analysis of proteins related to the JAK/STAT signaling pathway: JAK1, p‐JAK1, STAT1, p‐STAT1, STAT3, and p‐STAT3. (H) mRNA expression level of IFN‐γ in skin tissue detected by real‐time quantitative polymerase chain reaction (RT‐qPCR). (I, J) Positive expression of IFN‐γ in rat skin tissue detected by immunohistochemistry. Data are presented as the mean ± standard deviation (SD) from three independent experiments (*n* = 3).  ^∗^ *p* < 0.05,  ^∗∗^ *p* < 0.01,  ^∗∗∗^ *p* < 0.001,  ^∗∗∗∗^ *p* < 0.0001. AD, atopic dermatitis; CAP, capsaicin; IFN‐γ, interferon‐gamma; JAK1, Janus kinase 1; MOX, moxibustion; p‐JAK1, phosphorylated Janus kinase 1; p‐STAT1, phosphorylated signal transducer and activator of transcription 1; p‐STAT3, phosphorylated signal transducer and activator of transcription 3; STAT1, signal transducer and activator of transcription 1; STAT3, signal transducer and activator of transcription 3.

We further investigated the expression of the JAK/STAT signaling pathway: does moxibustion treatment for AD share a mechanism similar to capsaicin’s involvement in JAK/STAT signaling? Western blot results indicate that both moxibustion and capsaicin treatment in AD rats are associated with p‐JAK1, p‐STAT1, and p‐STAT3. Previous studies have demonstrated that IFN‐γ alleviates AD by reducing CLDN1 expression in keratinocytes through extracellular regulation of the transcription activator STAT3 and the JAK‐STAT1 signaling pathway [30, 31]. Therefore, we conducted an in‐depth analysis of the relative levels of IFN‐γ mRNA in the skin tissue and found that compared with the blank group, IFN‐γ expression levels were significantly elevated in the skin tissue of the AD rat model. Compared with the AD group, IFN‐γ expression levels were significantly reduced after moxibustion and capsaicin treatment. This result aligns with the expression level trends detected in the serum. It further validates that moxibustion inhibits inflammatory factor expression in AD treatment (Figure 5H,J). This indicates that moxibustion therapy, similar to capsaicin treatment, may exert its effects by reducing IFN‐γ levels and suppressing excessive activation of the JAK/STAT signaling pathway.

#### 3.3.4. Moxibustion and Capsaicin Were Associated With Changes in Pruritus‐Related Cutaneous Neuroimmune Mediators

To determine whether the observed reduction in scratching behavior was accompanied by changes in itch‐related molecular signals, serum IL‐31 and cutaneous IL‐31, TRPV1, substance P, and CGRP‐related markers were evaluated.

**Figure 6:**
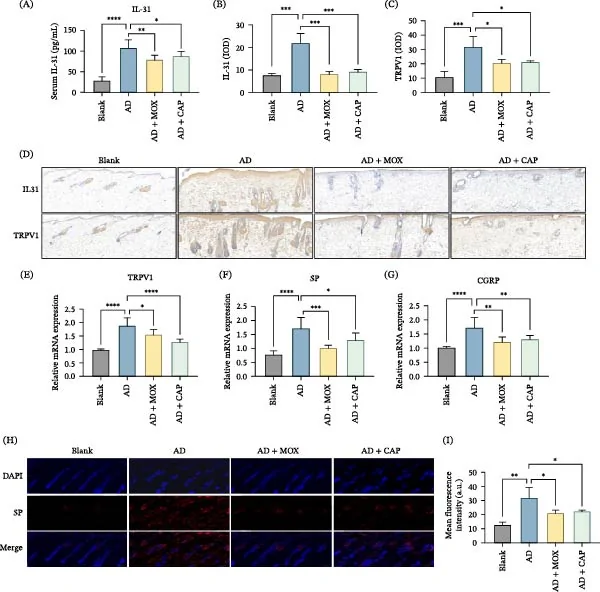
Effects of moxibustion and capsaicin on pruritus‐related neuroimmune mediators in AD rats. (A) Serum IL‐31 concentrations measured by ELISA (*n* = 6). (B) Quantitative analysis of cutaneous IL‐31 immunoreactivity (*n* = 3). (C) Quantitative analysis of cutaneous TRPV1 immunoreactivity (*n* = 3). (D) Representative immunohistochemical images of IL‐31 and TRPV1 in lesional skin. (E–G) Relative mRNA expression of Trpv1, SP(Tac1), and CGRP, respectively, in lesional skin. (H) Representative immunofluorescence images of substance P in lesional skin. Nuclei were counterstained with DAPI. (I) Quantitative analysis of substance P fluorescence (*n* = 3). Data are presented as the mean ± standard deviation (SD) from three independent experiments.  ^∗^ *p* < 0.05,  ^∗∗^ *p* < 0.01,  ^∗∗∗^ *p* < 0.001,  ^∗∗∗∗^ *p* < 0.0001. AD, atopic dermatitis; CAP, capsaicin; CGRP, calcitonin gene‐related peptide; IL‐31, interleukin 31; MOX, moxibustion; SP(TAC1), substance P (transcript: tachykinin precursor 1); TRPV1, transient receptor potential vanilloid 1. The term “CGRP” corresponds to α‐CGRP, which is encoded by the Calca gene.

Compared with the blank group, the untreated AD group showed a significant increase in serum IL‐31 concentration (Figure 6A). Immunohistochemical analysis also demonstrated increased IL‐31 and TRPV1 immunoreactivity in the lesional skin from the AD group (Figure 6B–D). Substance P immunofluorescence was significantly increased in the AD group relative to the control group (Figure 6H,I). RT‐qPCR analysis showed that lesional skin from the AD group had increased expression of Trpv1, SP, and CGRP relative to the control group (Figure 6E–G). Relative to the untreated AD group, moxibustion treatment significantly reduced serum IL‐31, cutaneous IL‐31, TRPV1, substance P, Trpv1, Tac1, and CGRP mRNA expression.

Taken together, these findings suggest that moxibustion alleviates scratching behavior, likely through modulation of an itch‐related neuroimmune signature involving IL‐31, TRPV1, substance P, and CGRP.

## 4. Discussion

The present study examined dermatitis and AD at two distinct but complementary levels. At the population level, the GBD analysis demonstrated that the aggregated dermatitis category continues to impose a substantial global burden and exhibits temporal, geographic, sex‐related, and socioeconomic heterogeneity. Because this GBD category includes atopic, contact, and seborrheic dermatitis, these findings cannot be attributed specifically to AD. They nevertheless provide a broader public‐health context for the continuing need to improve the management of chronic inflammatory and pruritic skin diseases. At the disease‐specific experimental level, DNCB exposure produced visible skin lesions, increased scratching behavior, histopathological injury, altered circulating inflammatory cytokines, and activation of itch‐related cutaneous neuroimmune markers. These findings are broadly consistent with recent DNCB‐induced dermatitis studies, in which increased lesion severity and inflammatory cytokine expression were accompanied by epidermal and dermal pathological changes [32, 33]. Moxibustion and capsaicin treatment were associated with improvements in lesion severity, scratching behavior, and histopathological changes. These effects were accompanied by reductions in inflammatory mediators, attenuation of selected IL‐31/TRPV1‐ and neuropeptide‐associated signals, and lower phosphorylation of JAK1, STAT1, and STAT3. These findings indicate that the treatment responses involved concurrent changes in inflammatory and sensory‐neuroimmune processes. Thus, the epidemiological and experimental components contribute evidence at different levels: the former characterizes the broader disease burden, whereas the latter provides disease‐specific evidence regarding therapeutic effects and potential mechanisms.

Capsaicin is a chemical agonist of TRPV1 and initially excites capsaicin‐sensitive sensory afferents, whereas repeated exposure may produce functional desensitization and reduced sensory responsiveness [12, 14]. Moxibustion, in contrast, delivers controlled physical heat and may additionally influence local blood flow, cellular stress responses, sensory activity, and immune regulation [15]. Experimental evidence indicates that some local tissue and immune responses induced by moxibustion can occur in TRPV1‐deficient animals, suggesting that the biological effects of moxibustion cannot be reduced to TRPV1 activation alone [34]. We therefore selected capsaicin not as a mechanistic surrogate for moxibustion but as an active comparator with a well‐characterized effect on peripheral sensory pathways. In the present study, cutaneous TRPV1 immunoreactivity and Trpv1 mRNA expression were increased in the untreated AD group relative to the healthy control group, consistent with an enhanced TRPV1‐associated pruritic environment in chronic AD‐like lesions. Both moxibustion and capsaicin reduced the elevated TRPV1 expression at the experimental endpoint. The reduction observed after capsaicin treatment does not imply that capsaicin acted as a TRPV1 antagonist. Acute channel activation and terminal receptor expression are distinct biological measures. Repeated capsaicin exposure can initially activate TRPV1 while subsequently promoting sensory‐afferent desensitization, receptor internalization, receptor degradation, or reduced responsiveness. In addition, our experiment evaluated IL‐31, substance P, and CGRP levels. Moxibustion‐associated reductions in these markers suggest that its antipruritic effect may involve the attenuation of communication among pruritogenic cytokines, epidermal cells, and peripheral sensory‐neuronal signals.

Sensory nerve endings, keratinocytes, and immune cells form a bidirectional communication network that regulates itch, neurogenic inflammation, barrier responses, and local immunity [13, 16, 17]. Physical heat and capsaicin represent distinct proximal stimuli, but both may alter this neurocutaneous network and generate partially convergent downstream inflammatory responses. In the present study, both treatments improved scratching behavior and inflammatory skin injury and were accompanied by overlapping proteomic alterations and reduced IFN‐γ/JAK/STAT pathway activity. These findings support the possibility of downstream neuroimmune and inflammatory convergence.

The thermal component of moxibustion provides one potential point of intersection with heat‐sensitive cutaneous sensory pathways. However, moxibustion is a complex local intervention [15, 34]. Modern research has found that temperatures during moxibustion can reach ~45°C. This form of stimulation is classified as a type of thermal stimulation. The effects of thermal stimulation on the immune system are multifactorial. Primarily, it enhances immune responses through the production and release of heat shock proteins (HSPs) [35, 36]. During thermal stress, increased release of HSPs into the extracellular environment stimulates downstream immune activity and enhances antigen presentation, subsequently activating T cells. HSPs participate in regulating both innate and adaptive immunity [37]. Furthermore, thermal stimulation has been shown to induce the release of related cytokines, which in turn enhances the immune surveillance function of T cells [38]. Previous studies have also indicated that moxibustion can modulate or enhance immune function while exerting anti‐inflammatory effects [39, 40]. In the present study, both the moxibustion and capsaicin groups showed a significant increasing trend in spleen and thymus indices. Concurrently, serum levels of the pro‐inflammatory cytokines IL‐1, IL‐4, TNF‐α, and IFN‐γ were significantly decreased, while the level of the anti‐inflammatory cytokine IL‐10 was increased. These findings collectively demonstrate that the mechanisms by which moxibustion and capsaicin alleviate AD are closely associated with immunomodulation.

AD is one of the diseases caused by an immune imbalance. Immune imbalance is the decisive factor leading to high environmental susceptibility and the occurrence of inflammatory responses in AD patients [19]. The pathogenesis of AD is closely associated with an imbalance between Th1 and Th2 cell responses. The acute phase is predominantly driven by Th2‐type reactions, whereas the chronic phase shifts to a Th1‐polarized dominance [41]. The Th1 cytokine IFN‐γ can contribute to epidermal barrier dysfunction by downregulating the expression of ceramides and long‐chain fatty acids [42]. During the transition from acute to chronic AD, dominant Th1 cells secrete cytokines such as IFN‐γ to suppress Th2‐mediated inflammation, manifesting clinically as skin thickening, hyperpigmentation, roughness, and lichenification. IFN‐γ levels are significantly elevated during chronic AD [43]. In chronic lesions, the activation of Th2 and Th22 cells is enhanced, while increased IFN‐γ production from Th1 cells further amplifies the inflammatory response. This cytokine can induce keratinocyte apoptosis and recruit more inflammatory cells to the skin, thereby perpetuating the itch‐scratch cycle and promoting lichenification [44]. In summary, IFN‐γ, a key mediator of Th1 immune responses, has been proven to be closely associated with chronic inflammation and pruritus in AD. Upon binding to its receptor IFN‐γR1, IFN‐γ induces receptor dimerization/polymerization, forming an activated receptor complex that subsequently activates JAK1. Phosphorylation of STAT proteins is also commonly induced by IFN‐γ [45]. Previous studies have concluded that IFN‐γ primarily exerts its effects via the JAK1, STAT1, and STAT3 signaling pathways, playing a pivotal role in inflammatory responses, macrophage activation, and Th1‐type immunity [46, 47]. Therefore, based on the findings of this study, we hypothesize that the common mechanism by which moxibustion and capsaicin alleviate pruritus in AD primarily involves therapeutic effects mediated through the inhibition of the JAK‐STAT signaling pathway regulated by the Th1 cytokine IFN‐γ.

The JAK‐STAT signaling pathway is a central component of the immune mechanism underlying AD pathogenesis [4, 48]. Inhibiting excessive activation of the JAK‐STAT signaling pathway can improve inflammation and pathological symptoms in AD [49, 50]. JAK inhibitors, such as upadacitinib, baricitinib, and abrocitinib, have been approved in multiple countries and regions for the treatment of moderate‐to‐severe AD. Their shared mechanism of action involves inhibiting the JAK‐STAT signaling pathway, thereby broadly attenuating the signaling of multiple key cytokines, including IL‐4, IL‐13, IL‐22, IL‐31, thymic stromal lymphopoietin, and IFN‐γ [51, 52]. Upon ligand binding to transmembrane cytokine receptors, such as interleukin or interferon receptors, JAK is activated and subsequently phosphorylates and activates STAT proteins. The activated STAT then translocates to the nucleus to regulate the transcription of specific target genes [5]. In mouse models of AD, overactivation of JAK1 induces spontaneous keratinocyte proliferation and STAT phosphorylation, driving skin barrier disruption and the development of pruritic dermatitis [48, 53]. In skin samples from AD patients, epidermal overphosphorylation of JAK1 [48] and upregulated IFN‐γ levels [54] have been detected. This aligns with the findings of the present study, which showed significantly elevated levels of both JAK1 and IFN‐γ in the model group. JAK1 is activated by IFN‐γ binding to its receptors, IFN‐γR1 and IFN‐γR2, leading to STAT1 phosphorylation. Phosphorylated STAT1 transduces the transcriptional signal into the nucleus, where it binds to the interferon‐γ‐activated sequence (GAS), thereby influencing IFN‐γ‐induced gene expression [55]. Within the nucleus, the STAT1 dimer binds to specific DNA sequences (GAS), initiating the transcription of pro‐inflammatory genes and amplifying inflammatory responses. In a mouse dry skin model, JAK inhibitors that block JAK1, JAK2, and JAK3 can improve skin barrier function by upregulating the expression of epidermal differentiation‐related proteins [56]. Other studies also support the notion that inhibiting the JAK/STAT pathway alleviates AD. For example, Igalan, a compound extracted from *Artemisia capillaris*, can suppress AD‐like responses in stimulated HaCaT keratinocytes via the JAK/STAT3 signaling pathway [57]. The experimental results of this study revealed that in the rat model of AD—a chronic, inflammatory, and recurrent disease—JAK/STAT signaling was abnormally activated during the disease course, accompanied by the overexpression of inflammatory factors such as IFN‐γ. However, after treatment with moxibustion and capsaicin, pathological features and itching symptoms showed significant improvement. Concurrently, both moxibustion and capsaicin suppressed the abnormal activation of JAK/STAT signaling and the overexpression of proinflammatory factors. Therefore, the mechanism by which moxibustion and capsaicin improve AD may be related to inhibiting the JAK/STAT signaling pathway and blocking immune crosstalk.

Several limitations should be acknowledged. First, the GBD component analyzed dermatitis as an aggregated Level 3 category comprising atopic, contact, and seborrheic dermatitis. Consequently, the observed epidemiological trends and socioeconomic inequalities cannot be attributed specifically to AD. Second, the population‐level and animal experimental components were designed to address different questions and were not analytically linked; the GBD findings provide contextual rather than mechanistic evidence. Third, although moxibustion is generally considered a relatively accessible nonpharmacological intervention, the present study did not conduct a formal cost‐effectiveness or healthcare‐accessibility analysis. Therefore, no conclusions regarding the economic superiority or population‐level effectiveness of moxibustion can be drawn from the current findings. Fourth, the exclusive use of female rats represents an additional limitation of the present study. Sex is an important biological variable in immune regulation and skin homeostasis, and experimental evidence indicates that sex hormones can influence local cutaneous immune responses. Because male rats were not included, the present study cannot determine whether disease severity, treatment responsiveness, inflammatory cytokine profiles, or JAK/STAT‐associated changes differ between sexes. Accordingly, the findings should be interpreted as being specific to female rats and should not be directly extrapolated to male animals. Future studies should include adequately powered groups of both sexes and formally examine sex‐by‐treatment interactions. Finally, although target intersection analysis, lesional‐skin proteomics, and measurements of IFN‐γ, p‐JAK1, p‐STAT1, and p‐STAT3 consistently implicated the JAK/STAT pathway, these complementary findings remain associative. The study did not include pharmacological inhibition or activation, genetic manipulation, or pathway‐rescue experiments. Therefore, it cannot establish whether JAK/STAT signaling is necessary or sufficient for the therapeutic effects of moxibustion and capsaicin, nor can it exclude the possibility that reduced pathway phosphorylation occurred secondarily to the overall improvement in cutaneous inflammation. Future studies incorporating JAK/STAT agonists or inhibitors, together with rescue experiments, will be required to determine the pathway causality. Accordingly, the present rat study provides preclinical evidence that moxibustion and topical capsaicin were associated with improvements in AD‐like skin lesions, scratching behavior, inflammatory activity, and selected itch‐related neuroimmune markers. The findings should be considered hypothesis‐generating and supportive of further translational investigation. Future studies should standardize the moxibustion procedure, incorporate sham‐moxibustion and formulation‐matched vehicle controls, evaluate dose–response relationships and local safety, and include longer follow‐up. Carefully designed clinical studies would then be required to assess efficacy, tolerability, acceptability, and durability using validated AD severity and pruritus outcomes.

## 5. Conclusion

In conclusion, the GBD 2021 analysis indicates that aggregated dermatitis remains a substantial global health concern, with heterogeneous temporal patterns and socioeconomic inequalities across regions. Because the GBD category includes atopic, contact, and seborrheic dermatitis, these population‐level findings should not be interpreted as specific estimates of AD burden. Within this broader public‐health context, the experimental findings demonstrate that moxibustion and topical capsaicin alleviate pruritus, inflammatory skin damage, and immune dysregulation in rats with DNCB‐induced AD. Their effects were accompanied by reduced IFN‐γ expression and suppression of JAK1, STAT1, and STAT3 phosphorylation, suggesting the potential involvement of the IFN‐γ/JAK/STAT axis. The epidemiological and experimental components provide complementary evidence at population and disease‐specific mechanistic levels, respectively, rather than a direct causal continuum.

## Author Contributions

Hong Zhao and Yunhong Yang contributed to the experimental design. Yunhong Yang, Yunsong Yang, Lihua Guo, and Hongjun Kuang did the statistical analysis. Yunsong Yang, Lihua Guo, Han Tang, Lvping Lin, Yi Gou, and Siyuan Li analyzed the data. Yunhong Yang wrote the manuscript. Hong Zhao revised the manuscript.

## Funding

This work was supported by the Shenzhen Science and Technology Program of China under Grant JCYJ20210324120804012, the National Natural Science Foundation of China under Grant 82474644, the Shenzhen Medical Research Fund under Grant C2501014, and the Sanming Project of Medicine in Shenzhen under Grant 202101007.

## Disclosure

To ensure the completeness and transparency of this animal study, the reporting of this research strictly adheres to the ARRIVE (Animal Research: Reporting of In Vivo Experiments) guidelines 2.0. We have completed the complete ARRIVE checklist and submitted it as Supporting Information alongside this manuscript. All authors reviewed the manuscript.

## Ethics Statement

This study was based on publicly available data from the GBD database and constitutes a noninterventional secondary data analysis. Since all data in the GBD database are aggregated, deidentified summary statistics at the population level and contain no identifiable private information, this research did not involve direct handling of human subjects or individual‐level data. In accordance with the World Medical Association’s Declaration of Helsinki and common ethical review guidelines, studies of this kind—which rely exclusively on publicly accessible, anonymized, and aggregated data—are generally eligible for exemption from ethical review. All data acquisition and analytical procedures in this study were conducted in full compliance with the terms of use of the GBD data.

The animal experimental procedures were approved by the Laboratory Animal Use and Management Committee of Guangzhou Huaten Bio‐Pharmaceutical Co., Ltd., IACUC Number: C202403‐6.

## Conflicts of Interest

The authors declare no conflicts of interest.

## Supporting Information

Additional supporting information can be found online in the Supporting Information section.

## Supporting information


**Supporting Information 1** Supporting Information: Table S2. Primer sequences.

**Supporting Information 2** Supporting Information: Table S1. Global and regional AAPCs in ASIR of dermatitis. AAPC, average annual percent change.

**Supporting Information 3** Supporting Information: Figure S1. Average annual percent changes of dermatitis. DALYs = disability‐adjusted life years; SDI = sociodemographic index. Error bars represent 95% uncertainty intervals. Positive values indicate an increasing trend, while negative values indicate a decreasing trend over the study period. Analyses were stratified by overall (Both), female, and male populations.

**Supporting Information 4** Supporting Information: Figure S2. Age‐standardized rate in dermatitis (per 100,000) DALYs = disability‐adjusted life years; SDI = sociodemographic index. Analyses were stratified by overall (Both), female, and male populations. The age‐standardized rates were computed using the Global Burden of Disease reference population to ensure comparability across regions and over time.

## Data Availability

The data that support the findings of this study are available from the the authors via the email address ginayyh@163.com upon reasonable request.
