# Supplementary material for: Population‐Level Burden of Dermatitis and the Effects of Moxibustion and Capsaicin on Pruritus and JAK/STAT Signaling in Atopic Dermatitis: A Multilevel Study
Source: Mediators Inflamm. 2026 Sep 16;2026:4932652. doi: 10.1155/mi/4932652 (PMC13580155; doi:10.1155/mi/4932652)
Supplement: Supplementary file 1 [file MI-2026-4932652-s002.docx]

| **Dermatitis** | | | |
| --- | --- | --- | --- |
| **Location** | **Period** | **AAPC (95% CI)** | **P-value** |
| Global | 1990-1999 | -0.117 (-0.123 to -0.111) | < 0.001 |
|  | 2000-2009 | 0.068 (0.067 to 0.069) | < 0.001 |
|  | 2010-2021 | 0.059 (0.054 to 0.063) | < 0.001 |
|  | 1990-2021 | 0.011 (0.008 to 0.013) | < 0.001 |
| High SDI | 1990-1999 | -0.899 (-0.933 to -0.865) | < 0.001 |
|  | 2000-2009 | 0.078 (0.073 to 0.082) | < 0.001 |
|  | 2010-2021 | 0.078 (0.073 to 0.082) | < 0.001 |
|  | 1990-2021 | -0.207 (-0.218 to -0.197) | < 0.001 |
| High-middle SDI | 1990-1999 | 0.111 (0.109 to 0.113) | < 0.001 |
|  | 2000-2009 | 0.082 (0.081 to 0.084) | < 0.001 |
|  | 2010-2021 | 0.031 (0.028 to 0.034) | < 0.001 |
|  | 1990-2021 | 0.072 (0.071 to 0.074) | < 0.001 |
| Middle SDI | 1990-1999 | -0.021 (-0.022 to -0.02) | < 0.001 |
|  | 2000-2009 | -0.023 (-0.023 to -0.023) | < 0.001 |
|  | 2010-2021 | -0.022 (-0.023 to -0.02) | < 0.001 |
|  | 1990-2021 | -0.022 (-0.023 to -0.021) | < 0.001 |
| Low-middle SDI | 1990-1999 | 0.013 (0.012 to 0.013) | < 0.001 |
|  | 2000-2009 | 0.005 (0.005 to 0.005) | < 0.001 |
|  | 2010-2021 | 0.013 (0.012 to 0.014) | < 0.001 |
|  | 1990-2021 | 0.01 (0.01 to 0.01) | < 0.001 |
| Low SDI | 1990-1999 | 0.001 (-0.005 to 0.006) | 0.857 |
|  | 2000-2009 | 0.004 (0.002 to 0.005) | < 0.001 |
|  | 2010-2021 | 0.005 (0.003 to 0.008) | < 0.001 |
|  | 1990-2021 | 0.003 (0.002 to 0.005) | < 0.001 |
