# Supplementary material for: Population‐Level Burden of Dermatitis and the Effects of Moxibustion and Capsaicin on Pruritus and JAK/STAT Signaling in Atopic Dermatitis: A Multilevel Study
Source: Mediators Inflamm. 2026 Sep 16;2026:4932652. doi: 10.1155/mi/4932652 (PMC13580155; doi:10.1155/mi/4932652)
Supplement: Supplementary file 2 [file MI-2026-4932652-s003.docx]

| **Gene** | **Primer sequence (5′→3′)** |
| --- | --- |
| β-actin (rat) | F: CCGCGAGTACAACCTTCTTG |
|  | R: TGACCCATACCCACCATCAC |
| IFN-γ(rat) | F: GTTCGAGGTGAACAACCCAC |
|  | R: CAGAATCAGCACCGACTCCTT |
| TRPV1(rat) | F: TGACAGCGAGTTCAAAGACC |
|  | R: CTGGCATTGACAAACTGCTT |
| SP(rat) | F: ACCAACACTTCAGAACCCAACC |
|  | R: AACAGACCGTAGTACCACTCA |
| CGRP(rat) | F: GTTGGCTATTGTGCATCGTGTT3 |
|  | R: CCGCTTGAGGTTTAGCAGAGTT |
